# Supplementary material for: A highly utilized and practical lithium-sulfur positive electrode enabled in all-solid-state batteries
Source: Nat Commun. 2026 Feb 27;17:3298. doi: 10.1038/s41467-026-69750-0 (PMC13066583; doi:10.1038/s41467-026-69750-0)
Supplement: Supplementary file 1 — Supplementary Information [file 41467_2026_69750_MOESM1_ESM.pdf]

Supplementary Information

A highly utilized and practical lithium-sulfur positive electrode enabled in all-solid-state batteries

Ashley Cronk[1], Xiaowei Wang[2]†, Jin An Sam Oh[2], So-Yeon Ham[1], Shuang Bai[1], Phillip Ridley[2], Mehdi Chouchane[3], Chen-Jui Huang[3], Diyi Cheng[2], Grayson Deysher[1], Hedi Yang[3], Baharak Sayahpour[1], Marta Vicencio[2], Choonghyeon Lee[4], Dongchan Lee[4], Min-Sang Song[4], Jihyun Jang[2]‡, Jeong Beom Lee[4*], and Ying Shirley Meng[2,3*]

The PDF file includes:

Figures S1 to S35
Tables S1 to S8

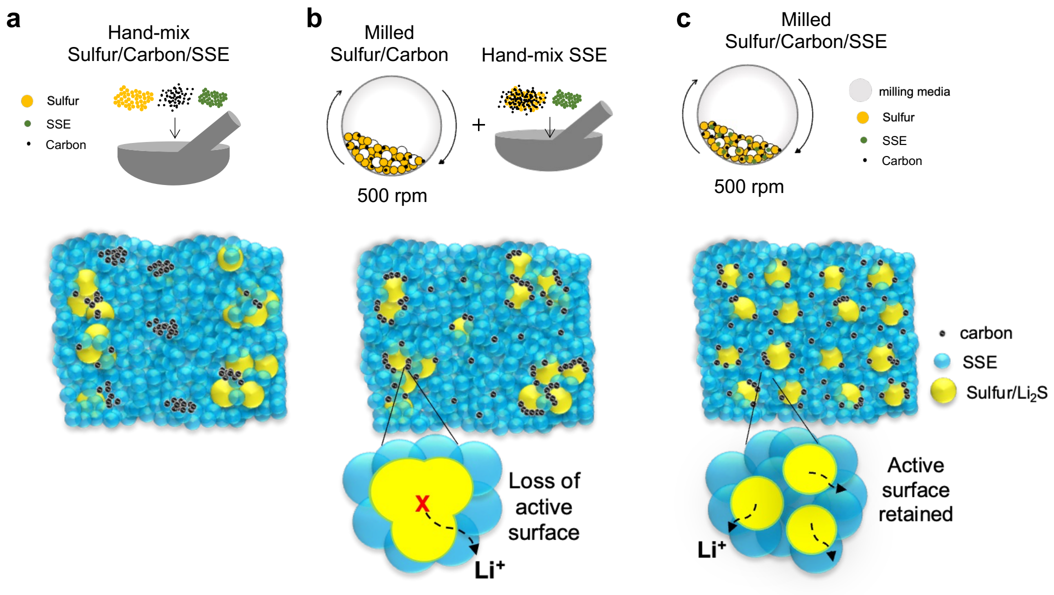

Fig. S1. Various positive electrode composite synthesis methods. Schematic illustrating the process of (a) hand-mixing all components, (b) milling carbon and sulfur followed by hand-mixing SSE and (c) single step milling of all components with their expected distribution based on the 1st formation cycle.

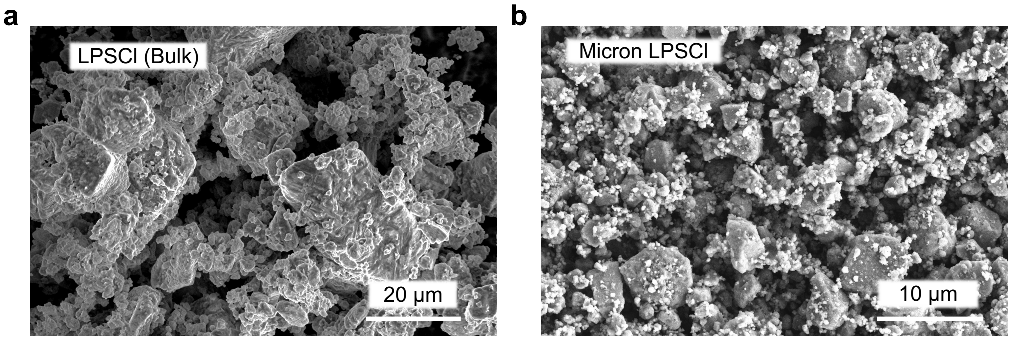

Fig. S2. SEM images of LPSCl solid electrolyte particles (a) as received and (b) after milling to reduce particle size to the micron scale. *Protocols are included within the methods section.*

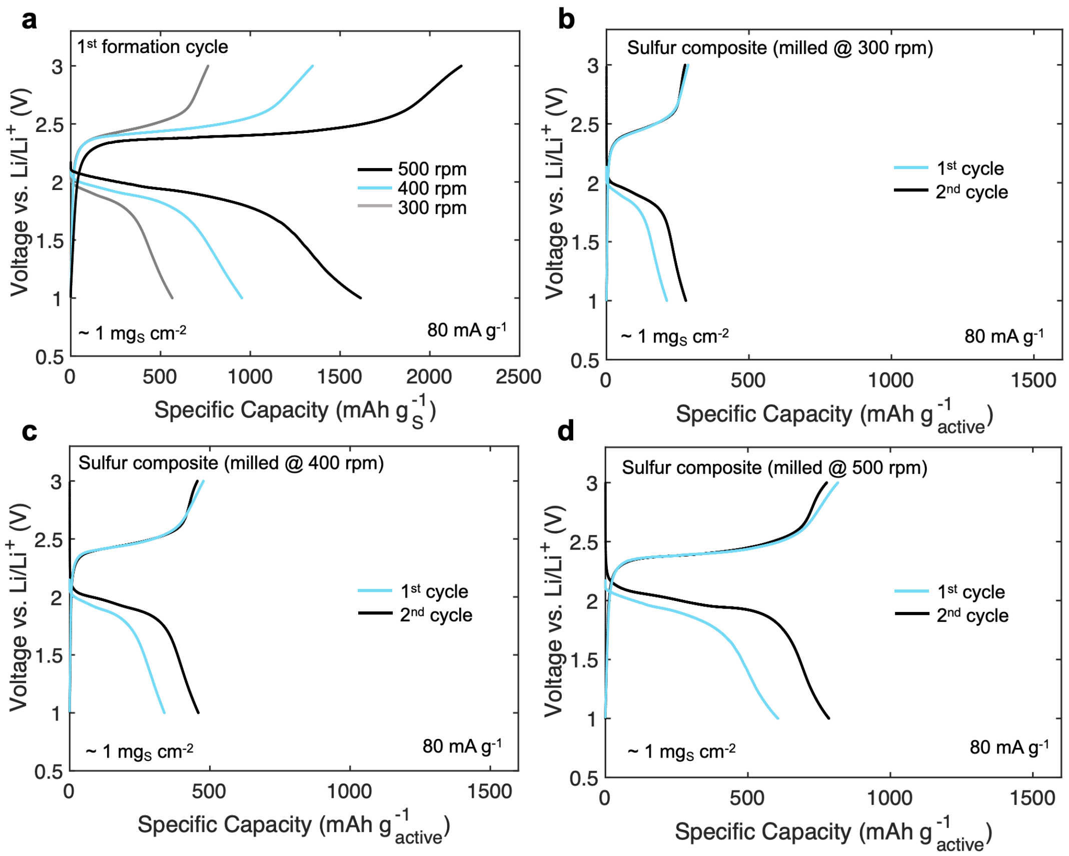

Fig S3. Evaluation of lower milling intensities using the one-step milling process in Li-In half cells. (a) 1st formation cycle normalized by sulfur mass. Voltage profiles normalized by total active mass (sulfur and LPSCl) for composites fabricated at (b) 300 rpm, (c) 400 rpm, and (d) 500 rpm. Stack pressure: 75 MPa. Testing Temperature: 25°C ± 1°C.

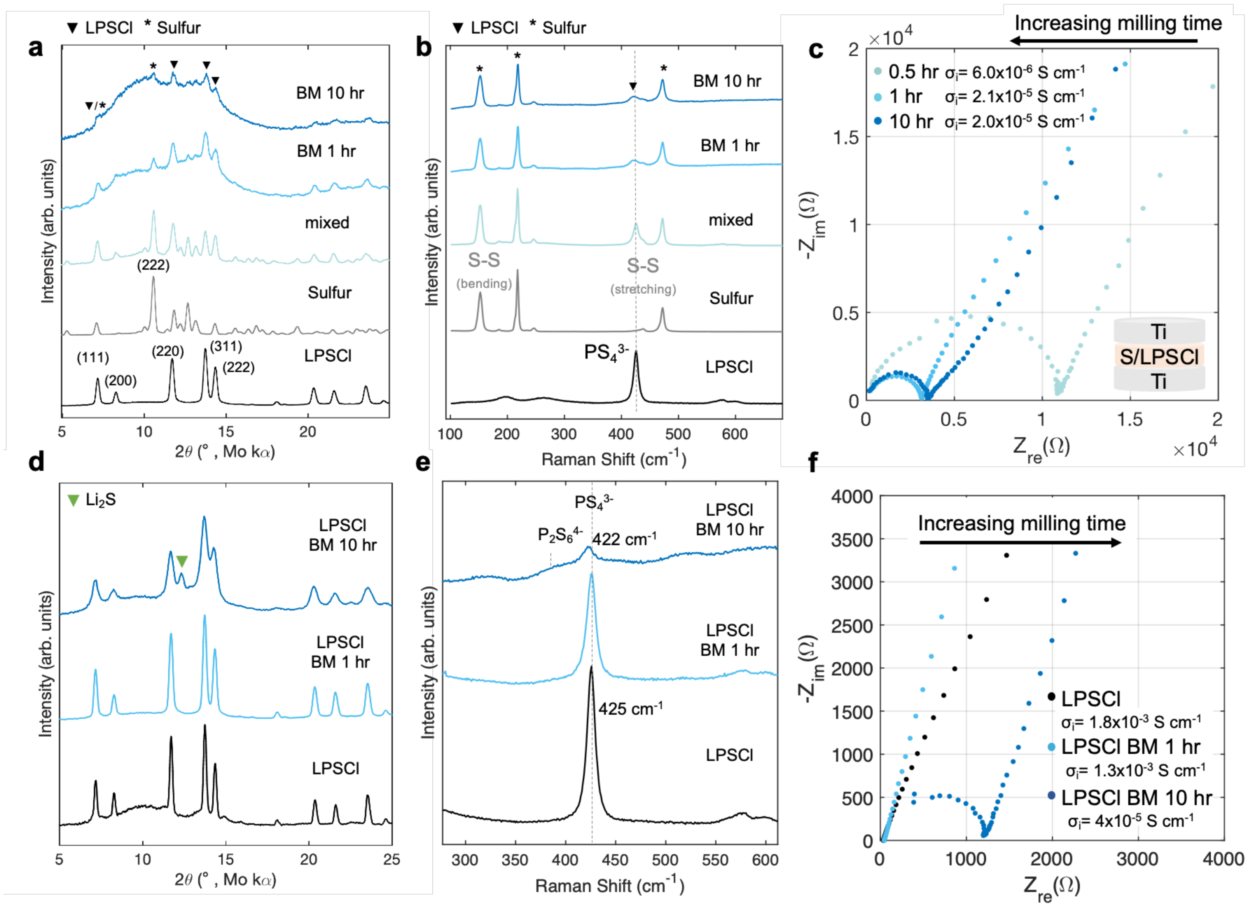

Fig. S4. Characterization of sulfur and LPSCl catholyte after one-step synthesis without carbon. a, XRD spectra of S/LPSCl composite with increased milling durations. **b**, Raman spectra of composites shown in a. **c**, Corresponding Nyquist plots of S/LPSCl composites with increasing milling durations. Standalone **d**, XRD spectra **e**, Raman spectra, and **f**, Nyquist plots of LPSCl after 1 hour and 10 hour milling durations. Stack pressure: 75 MPa. Testing Temperature: 25°C ± 1°C.

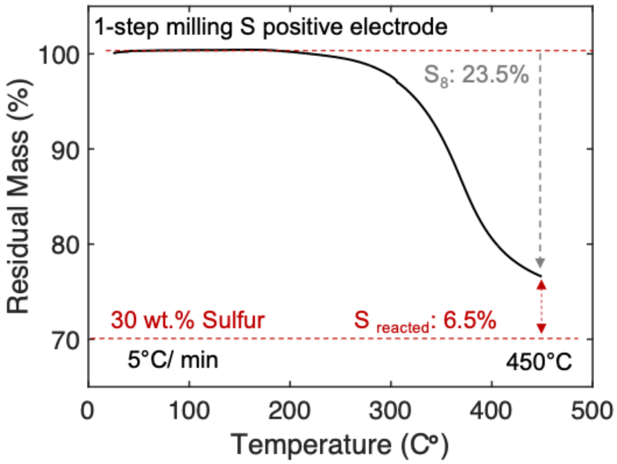

Fig. S5. Thermogravimetric analysis (TGA) of the sulfur positive electrode composite evaluated with a thermal rate of 5 C°/min in nitrogen atmosphere.

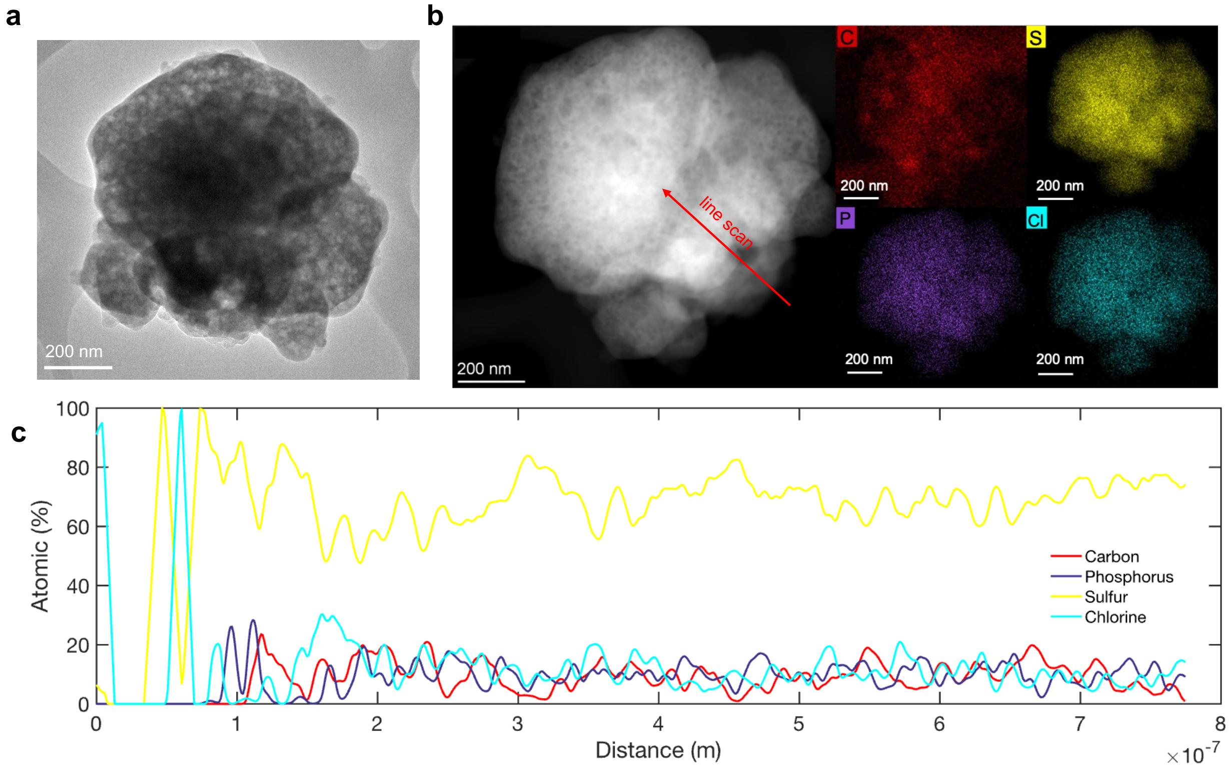

Fig. S6. **Analysis of sulfur positive electrode particles after one-step synthesis.** **a**, Low magnification TEM image. **b**, HAADF-STEM image of sulfur/LPSCl/C agglomerated particle 1 after synthesis with EDS hyperspectral imaging, and (**c**) line scan results.

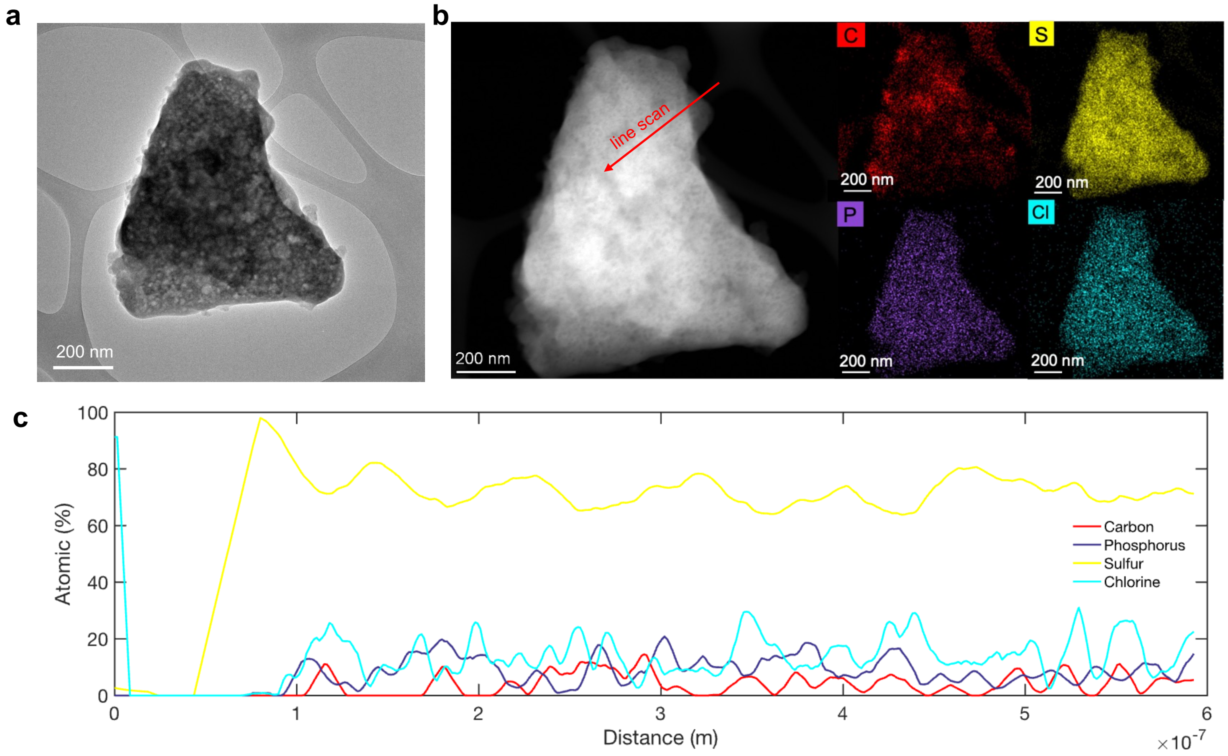

Fig. S7. Analysis of sulfur/LPSCl/C particle 2 after one-step synthesis. **a**, Low magnification TEM image. **b**, HAADF-STEM image of sulfur/LPSCl/C agglomerated particle 2 after synthesis with EDS hyperspectral imaging, and (**c**) line scan results.

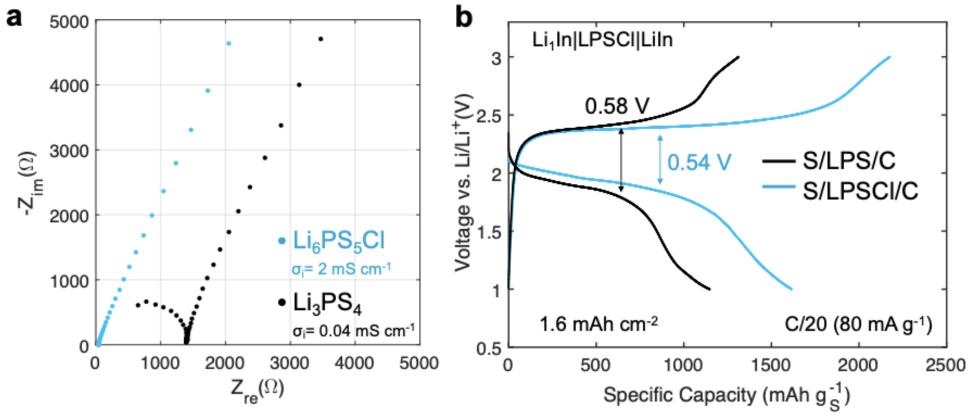

Fig. S8. Performance comparison with LPS and LPSCl catholytes within sulfur positive electrodes. (a) Nyquist plots of EIS measurements comparing LPSCl and LPS. (b) Electrochemical performance of sulfur positive electrodes using the 1-step milled synthesis approach fabricated with either LPSCl or LPS as the catholyte. Half cells were fabricated with a Li-In negative electrode. Stack pressure: 75 MPa. Testing Temperature: 25°C ± 1°C.

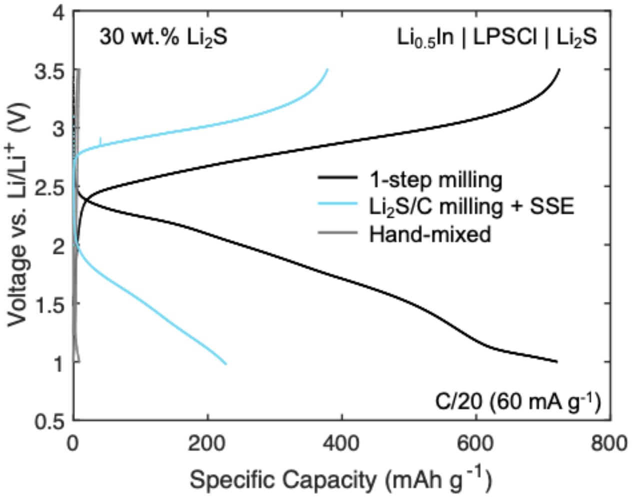

Fig. S9. 1st formation cycle voltage profiles of half cells consisting of Li_2S positive electrodes prepared by various methods. Stack pressure: 75 MPa. Testing Temperature: 25°C ± 1°C.

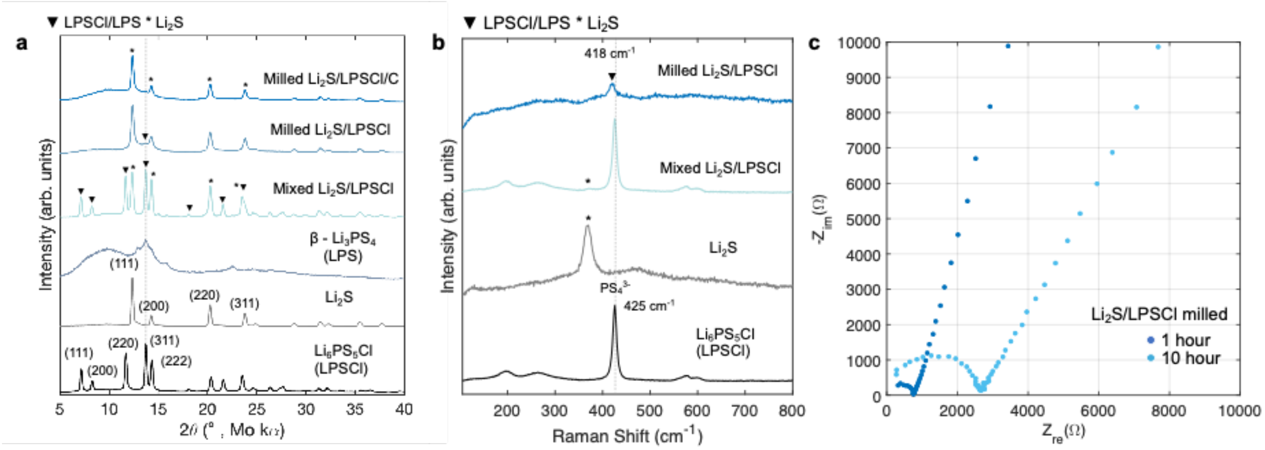

Fig. S10. Characterization of the Li_2S positive electrode. a, X-ray diffraction and b, Raman spectroscopy spectra comparing milled and mixed Li_2S/LPSCl composites without carbon. c, Nyquist plots of Li_2S/LPSCl composites after 1 and 10 hours of milling. For the EIS measurements the stack pressure was 75 MPa. Testing Temperature: 25°C ± 1°C.

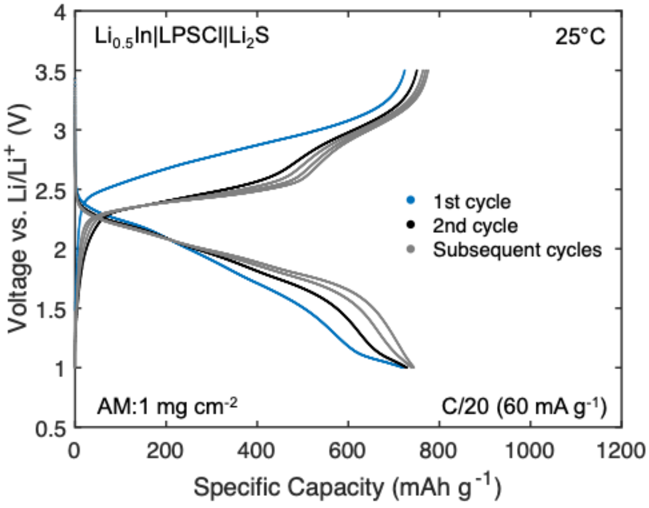

Fig. S11. Voltage profiles of a $Li_{0.5}In$|LPSCl|Li_2S cell evaluated at 25°C at C/20 cycling conditions (60 mA g^{-1}). Stack pressure: 75 MPa. Testing Temperature: 25°C ± 1°C.

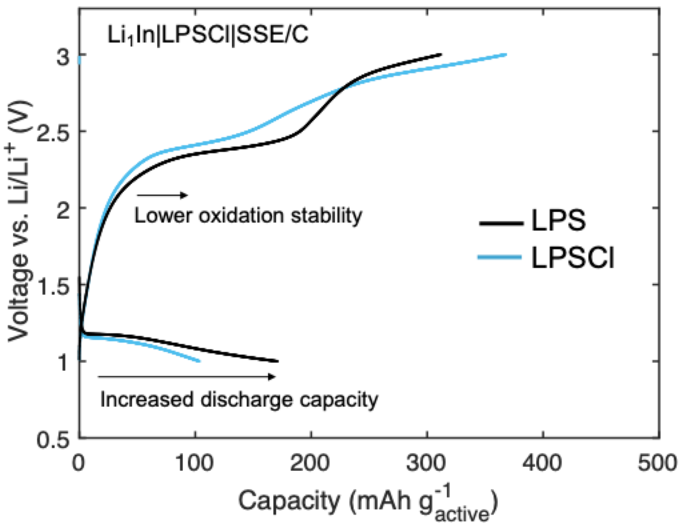

Fig. S12. 1st cycle voltage profiles of LPS and LPSCl (80 wt.% SSE and 20 wt.% carbon) as positive electrodes evaluated in Li-In half cells. These cells were cycled under a rate of 0.02 A g^{-1}, which is approximately C/20. Stack pressure: 75 MPa. Testing Temperature: 25°C ± 1°C.

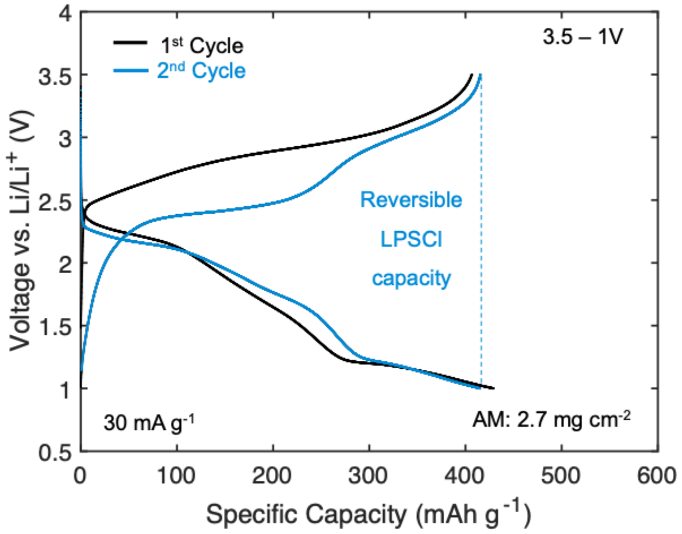

Fig. S13. Voltage profiles of a Li-In half cell using just LPSCl as the positive electrode (80 wt.% SSE and 20 wt.% carbon) evaluated within Li_2S voltage limits from 3.5V to 1V. Stack pressure: 75 MPa. Testing Temperature: 25°C ± 1°C.

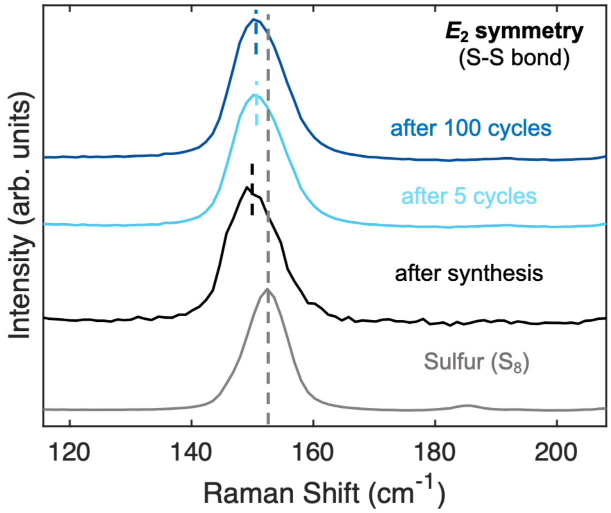

Fig. S14. Raman spectra of cycled sulfur positive electrodes focusing on the symmetrical S-S bending region.

a

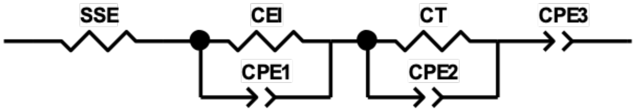

b

Composite	State	Resistance (Ω)			Capacitance (10^{-3} F)		χ^2 (10^{-3})
		SSE/Bulk	CEI	CT	CEI	CT	
S positive electrode	Pristine (OCV)	32.9	22.5	/	0.030	/	4.1
	Discharged (1V)	34.7	24.6	85.2	0.002	7.2	2.5
	Charged (3V)	30.1	28.5	/	0.920	/	3.1

Fig. S15. (a) Equivalent circuit used for fitting EIS spectra in Fig. 4a. (b) Fit results.

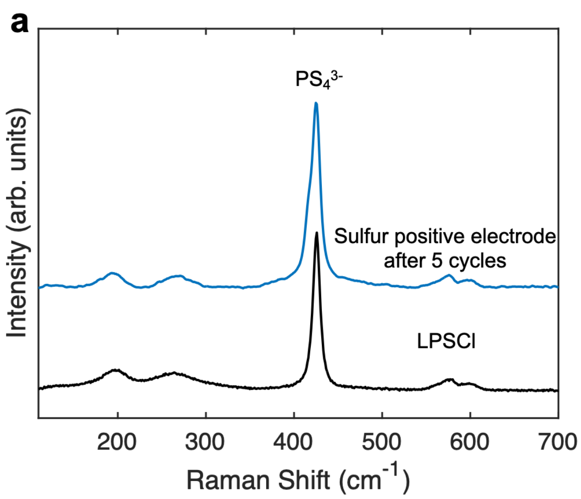

Fig. S16. Raman spectra of the cycled sulfur positive electrode at a region correlating within the SSE bulk. (a) Comparison to pristine LPSCl and (b) fitted spectra highlighting two different PS_4^{3-} phases attributed to LPSCl and LPS.

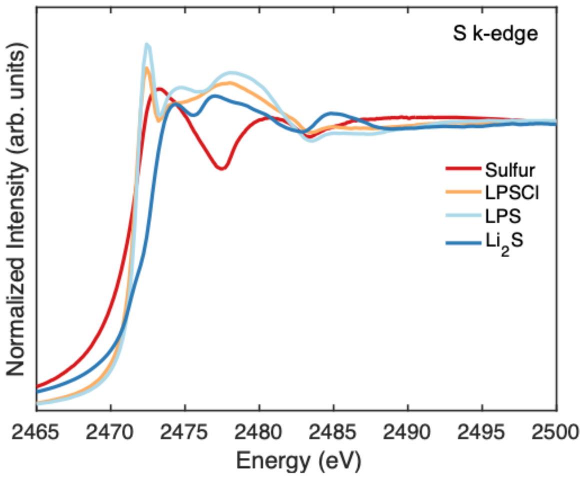

Fig. S17. Sulfur K-edge X-ray absorption spectroscopy (XAS) spectra of reference species and expected redox products sulfur positive electrode redox products.

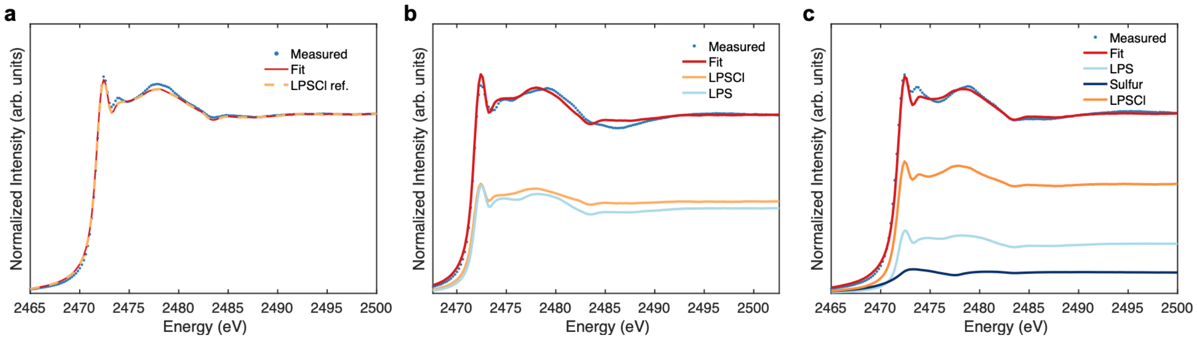

Fig. S18. Linear combination fitting of XAS spectra collected at the Sulfur K-edge of the LPSCl positive electrode system for the (**a**) pristine, (**b**) discharged state, and (**c**) charged (after 1 cycle) state.

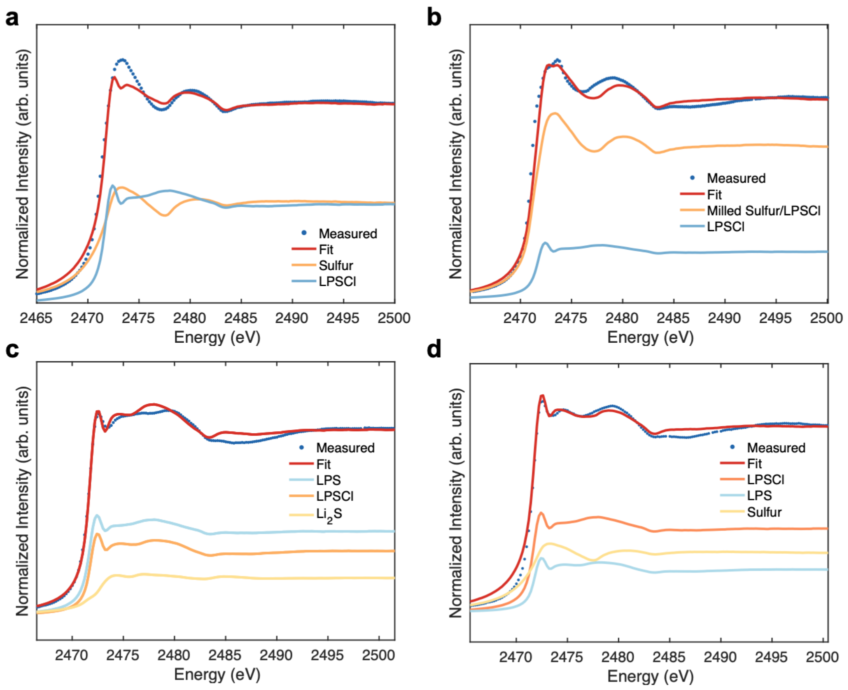

Fig. S19. Linear combination fitting of XAS spectra at Sulfur K-edge of the sulfur system. a, Milled Sulfur/LPSCl (without carbon). **b**, Pristine composite. **c**, Discharged composite. **d**, Charged composite (after 1 cycle) conditions.

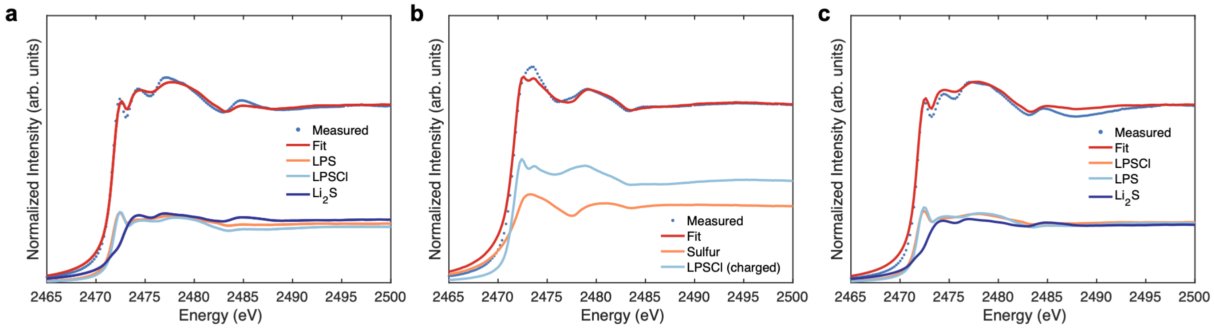

Fig. S20. Linear combination fitting of XAS spectra at Sulfur k-edge of the Li_2S system. (**a**) pristine composite and under (**b**) charged and (**c**) discharged (after 1 cycle) conditions.

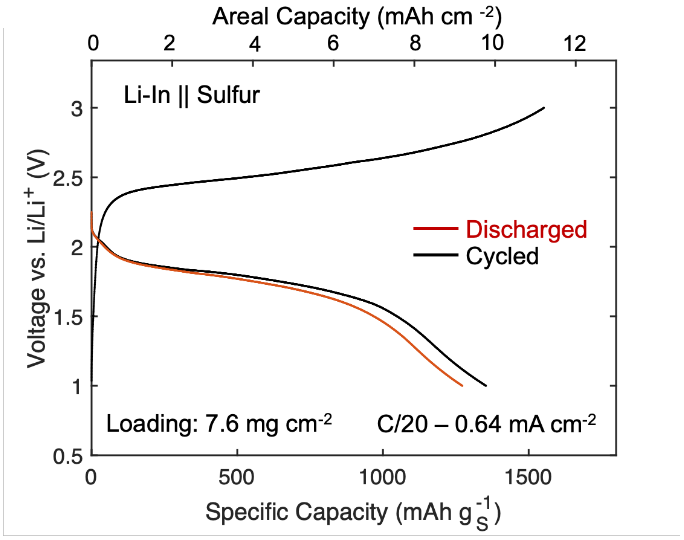

Fig. S21. Electrochemical performance of sulfur positive electrodes used for XRD analysis.

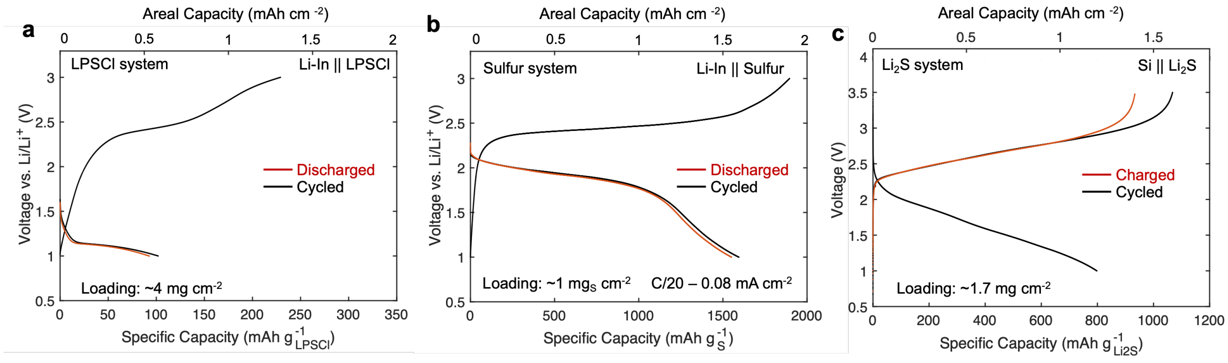

Fig. S22. Electrochemical performance of cells with (a) LPSCl, (b) sulfur, and (c) Li_2S positive electrodes used for XANES quantification.

Quantification limitations of XANES at the Sulfur K-edge

XANES was performed in total fluorescence yield (TFY) mode. In this mode, sulfur spectra can distort due to its strong self-absorption effect.[1] X-ray energies for the Sulfur K-edge are low (2keV) as compared to hard X-rays (5-10 keV), resulting in a limited probe depth of 500 nm to several microns. This narrows the detection region to particle surfaces and their interfaces. The beam spot size for the beamline used in this work is 0.25 mm × 0.16 mm, which covers 0.06% of the electrode area. Therefore, collecting information on the entire positive electrode can be challenging. This is why XRD was conducted in parallel.

XANES quantification was performed using linear combination fitting (LCF) to identify and quantify overlapping redox products between sulfur, LPSCl, and Li_2S. LCF deconvolutes the relative proportions of different references within the sample of interest, assuming the composite spectra are a linear combination of individual components. Quantification software such as Athena (used in this work) performs non-linear least squares minimization to minimize the error between measured and fitted spectra. This fitted spectra is a linear combination of the calculated weights, each corresponding to a specific reference spectrum. Weight uncertainties can be introduced from noise and when the reference spectra exhibit similar features (like LPSCl and LPS), where both possess the thiophosphate unit (PS_4^{3-}) within their structure. These factors were considered in our analysis.

Supplementary References

1. F. Jalilehvand. Sulfur: not a "silent" element anymore. *Chem. Soc. Rev.,* 2006, 35, 1256–1268.

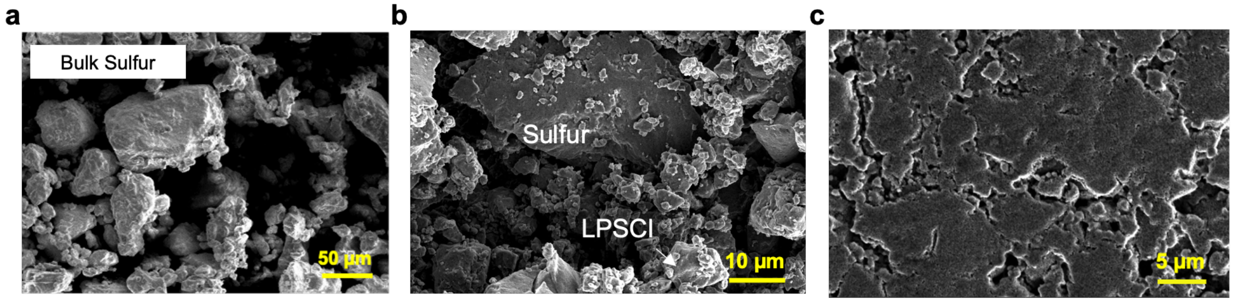

Fig. S23. Scanning electron microscopy (SEM) images of unmodified sulfur and composite. Powders of (a) Bulk Sulfur as received and (b) composite after synthesis. (c) Top view after positive electrode composite fabrication.

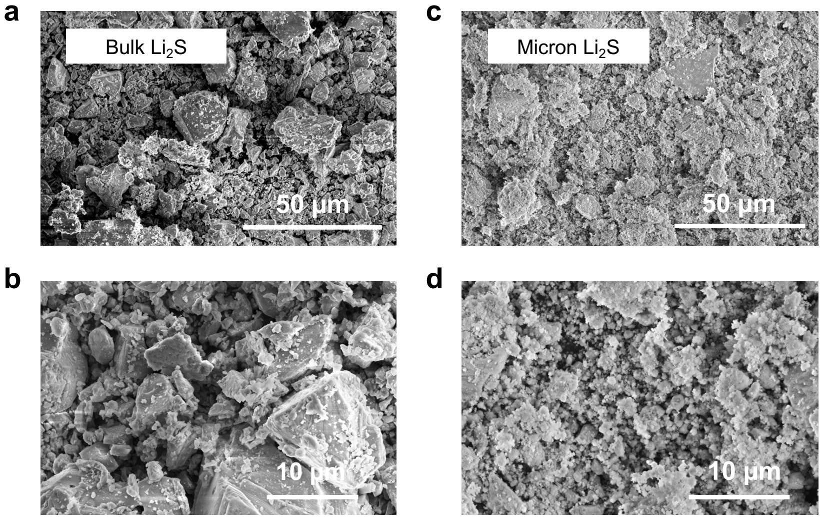

Fig. S24. SEM images of Li_2S powders. **a**, Low magnification and (**b**) high magnification of as received bulk Li_2S particles. **c**, Low magnification and (**d**) high magnification of micron Li_2S particles.

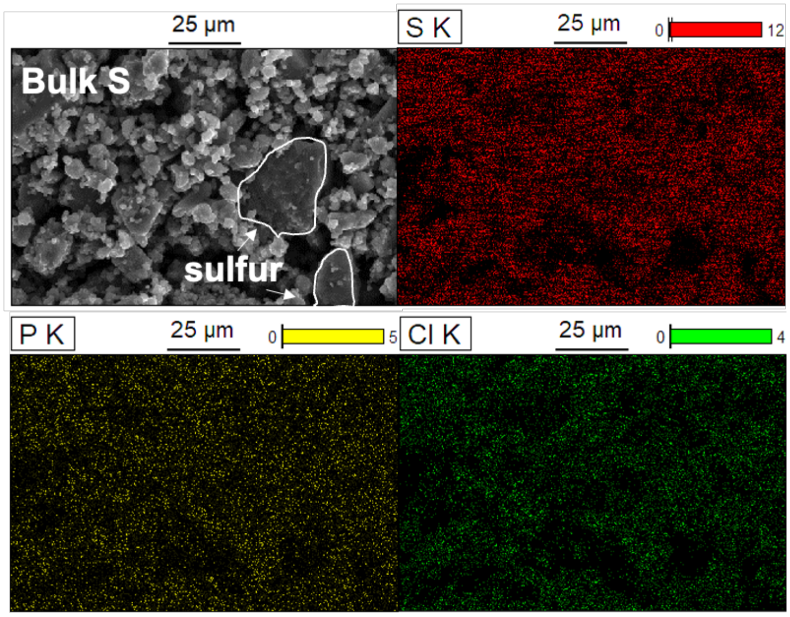

Fig S25. SEM-EDS mapping of the sulfur positive electrode composite powders synthesized with unmodified Bulk Sulfur.

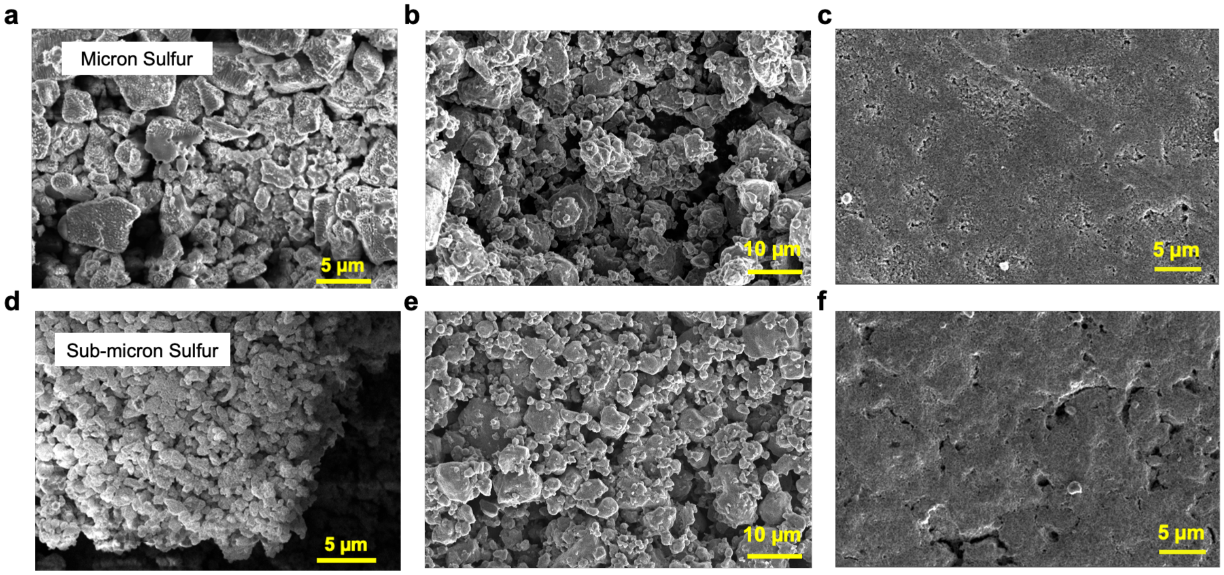

Fig. S26. Scanning electron microscopy (SEM) images of milled sulfur particles and their composites. Powders of (a) Micron scale sulfur, (b) composite after synthesis, and (c) top view after positive electrode composite fabrication. Powders of (d) Sub-micron scale sulfur, (e) composite after synthesis, and (f) top view after positive electrode fabrication.

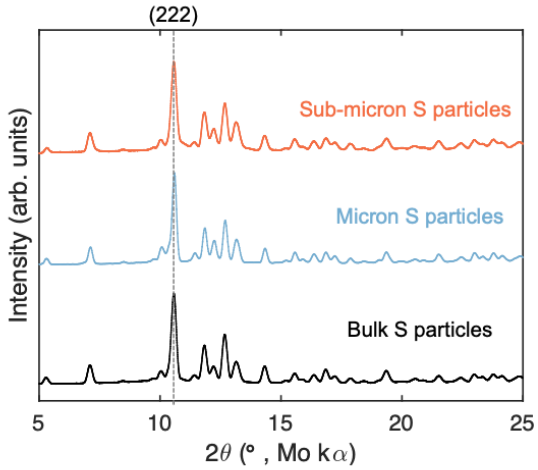

Fig. S27. XRD spectra of sulfur particles after particle size reduction to the micron and sub-micron scale.

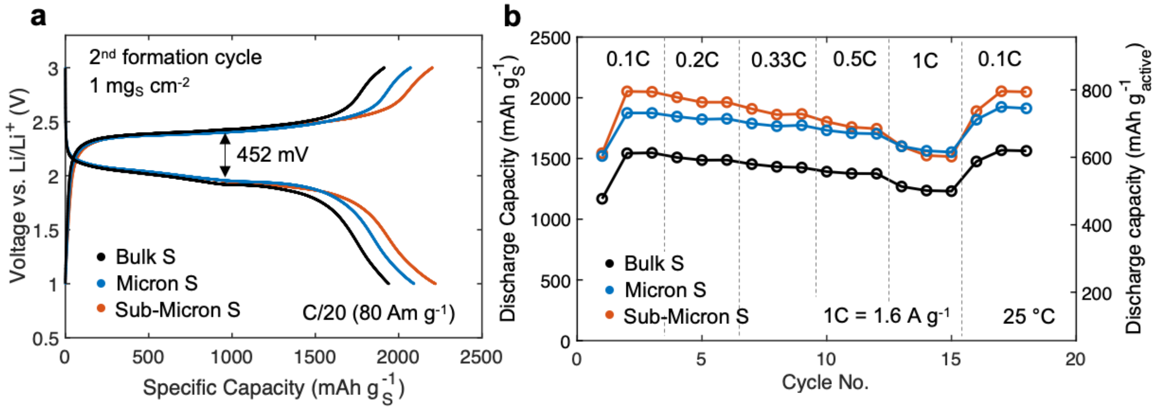

Fig. S28. Electrochemical results with differing sulfur particle sizes. **a**, Second formation cycle of half cells constructed with bulk, micron, and sub-micron sulfur composites and a Li-In negative electrode. **b**, Rate performance evaluation from 0.1C to 1C at 25°C, normalized by both sulfur (left y-axis) and active materials (S/LPSCl) (right y-axis).

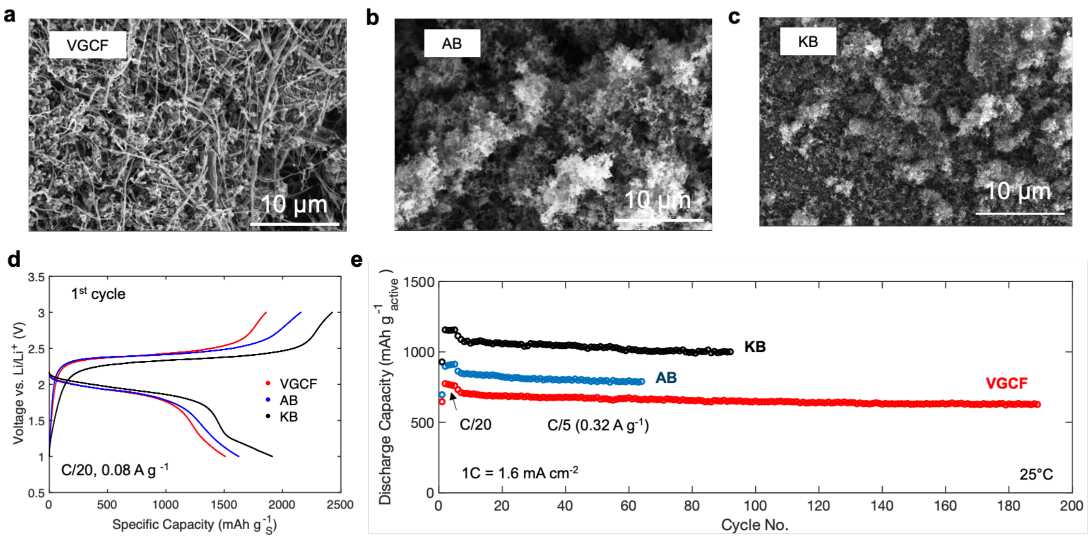

Fig. S29. Electrochemical evaluation of the micron sulfur positive electrode Li-In half cells with various carbon types. SEM images of (**a**) vapor grown carbon fiber (VGCF), (**b**) acetylene black (AB) and (**c**) Ketjen black (KB, EC-600JD). (**d**), First cycle voltage profile at C/20. (**e**), Long term cycling performance at C/5.

a

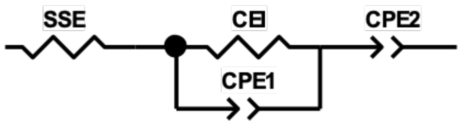

b

Composite	Cycle	Resistance		Capacitance (10^{-3} F)	χ^2 (10^{-3})
		SSE/Bulk	CEI	CEI	
Micron S	100	20.7	260.9	2.3	0.8
	300	18.3	284.6	2.5	0.4
	500	14.9	357.7	4.5	1.0
Sub-micron S	100	34.9	416.9	3.9	0.9
	300	29.2	443.0	4.1	0.5
	500	30.4	511.8	10	1.2

Fig. S30. (a) Equivalent circuit used for fitting EIS spectra in Fig. 5f. (b) Fit results.

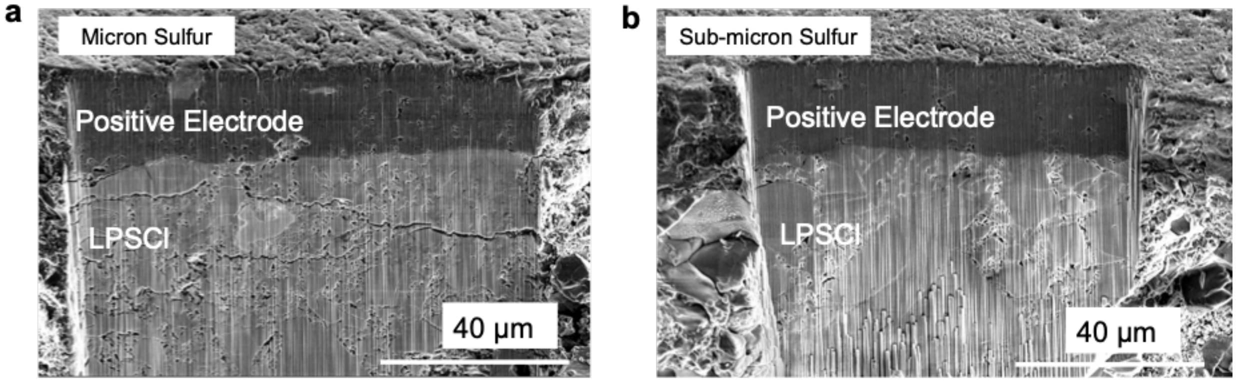

Fig. S31. Cross-sectional SEM images of sulfur positive electrode and SSE layer under cryogenic conditions. **a**, Micron sulfur and (**b**) Sub-micron sulfur positive electrodes after fabrication.

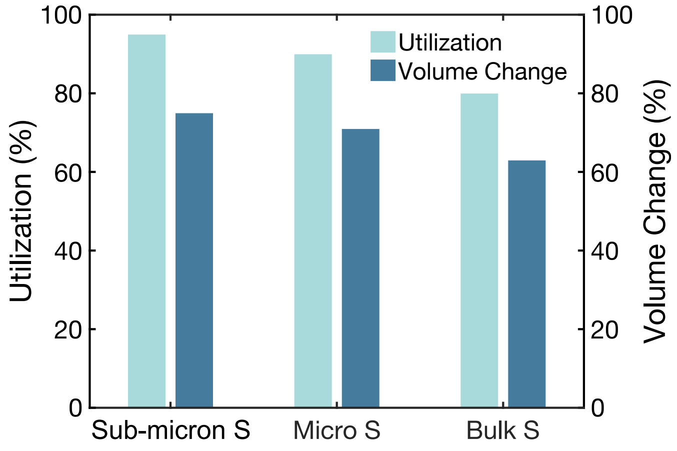

Fig. S32. Estimated volume expansion of sulfur particles in positive electrodes based on % utilization obtained from first discharge capacities at C/20 (80 mA g^{-1}). This estimation also considers the capacity contribution of LPSCl during the discharge process.

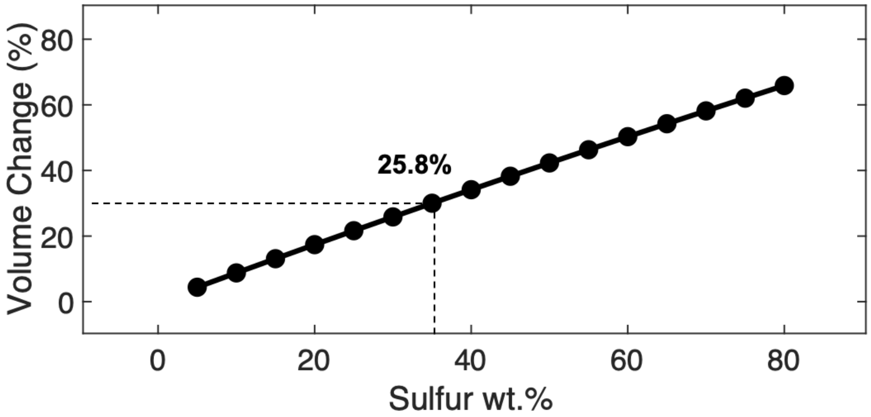

Fig. S33. Estimated volume change (%) of the sulfur positive electrodes as a function of sulfur weight percent. Assuming only sulfur undergoes 80% volume change after complete lithiation.

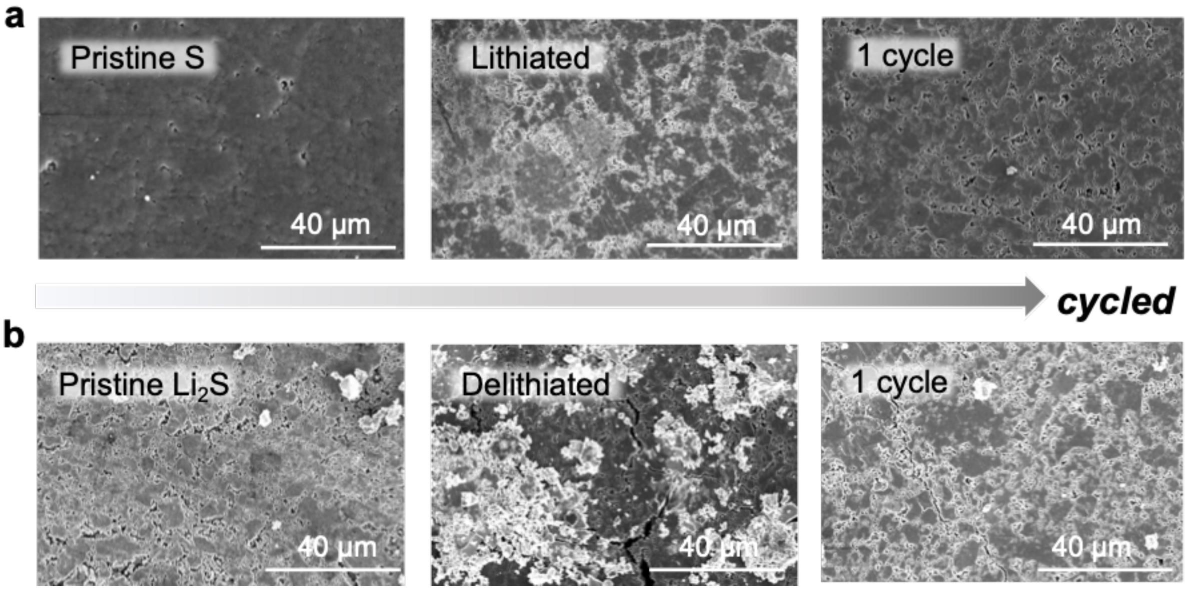

Fig. S34. Top surface images of Li-S positive electrodes at various states of charge. **a**, Sulfur positive electrode and (**b**) Li_2S positive electrode composite surface morphology with cycling.

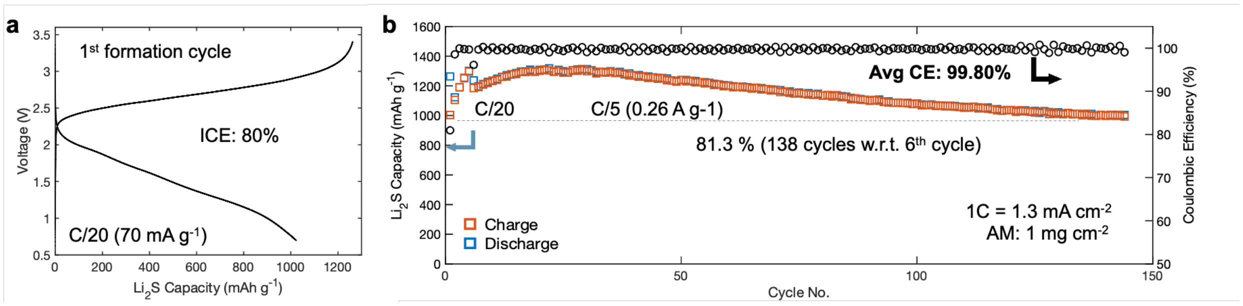

Fig. S35. Full cell electrochemical performance consisting of Li_2S positive electrode and Si negative electrode evaluated at 25°C. **a**, First cycle voltage profile. **b**, Long term cycling stability of five C/20 (70 mA g^{-1}) cycles with subsequent cycles at C/5 (0.26 A g^{-1}). N/P ratio: 2.

Table S1. Scanning transmission electron (STEM) microscopy elemental mapping results.

Particle 1

Z	Element	Family	Atomic Fraction (%)	Atomic Error (%)	Mass Fraction (%)	Mass Error (%)	Fit Error (%)
8	O	K	16.65	3.94	9.02	1.27	0.85
15	P	K	7.82	2.37	8.20	1.94	0.95
16	S	K	68.68	20.67	74.56	17.36	0.12
17	Cl	K	6.85	2.05	8.22	1.89	0.56

Particle 2

Z	Element	Family	Atomic Fraction (%)	Atomic Error (%)	Mass Fraction (%)	Mass Error (%)	Fit Error (%)
8	O	K	19.88	4.57	10.96	1.50	0.29
15	P	K	7.62	2.27	8.14	1.90	0.58
16	S	K	65.79	19.44	72.70	16.78	0.19
17	Cl	K	6.71	1.97	8.20	1.87	0.28

Table S2. Estimated capacity contributions between unreacted sulfur, reacted sulfur in Li3PS$_{4+n}$, and LPSCl based on a total sulfur positive electrode mass of 2.5 mg with a composition of 30 wt.% sulfur, 50 wt.% LPSCl, and 20 wt.% carbon.

Expected Capacity Based on Mass for each Component				
	Unreacted Sulfur (mAh)	Reacted Sulfur in Li_3PS_{4+n} (mAh)	LPSCl (mAh)	Total Capacity (mAh)
Discharge	1	0.25	0.13	1.38
Charge	1	0.25	0.44	1.70
Capacity Estimated based on Electrochemistry				
Discharge	1	0.20		1.20
Charge	1	0.16	0.44	1.60
Capacity Estimated based on potentials from dQ/dV				
Discharge	1	0.09	0.11	1.20
Charge	1	0.16	0.44	1.60
Capacity Contributions (%)				
Discharge	83.3	7.5	9.2	1.20
Charge	62.1	10	27.5	1.60

Table S3. Estimated capacity and sulfur utilization in positive electrodes from cells shown in Fig. 3c.

**Sulfur wt. %**	**LPSCl wt. %**	**Active wt.%**	**Discharge (mAh)**	**Charge (mAh)**	**mAh g_{sulfur} (Without subtracting LPSCl)**	**LPSCl capacity (mAh)**	**Sulfur capacity (mAh)**	**mAh g_{sulfur} (Subtracting LPSCl capacity)**	**Sulfur utilizatio n (%)**
0	80	80	0.14	0.46	-	-	-	-	-
10	70	80	0.60	1.19	1900	0.22	0.37	1543	92
22	55	77	1.15	1.71	1647	0.22	0.93	1411	84.3
30	50	80	1.20	1.63	1615	0.14	1.05	1408	84

Table S4. Sulfur positive electrode samples from Li-In || sulfur half cells with an areal capacity of 10 mAh cm^{-2} prepared for TGA measurements at different states of charge.

Electrode Sample	Positive Electrode Mass (mg)	Sulfur Mass (mg)	TGA Mass Loss (%)	Sulfur detected with TGA (mg)	Sulfur Mass – Sulfur detected with TGA
Pristine	18.7	5.61	23.7	4.43	1.2 mg / 20 % reacted
Discharged	19.8	5.94	2.1	0.41	5.53 mg / 93.1 % reacted
Charged	14.1	4.23	19.7	2.77	1.5 mg / 34% reacted

Table S5. Sulfur K-edge XAS linear combination fitting results.

Composite	Sample	Species Wt.%				χ^2	R-factor
		Sulfur	LPSCl	LPS	Li_2S		
LPSCl/C	Pristine	0	100	0	0	0.03044	0.0005281
	Discharge	0	52.1	47.9	0	0.06229	0.0010326
	Charge	11.60	60.9	27.5	0	0.07749	0.0012868
S/LPSCl/C	Pristine	38.37	61.6	0	0	0.24154	0.0041802
	Discharge	0	45.3	34.4	20.2	0.0983	0.0017195
	Charge	32.0	45.1	22.9	0	0.05798	0.0219914
Li_2S/LPSCl/C	Pristine	0	33.2	31.2	35.6	0.04966	0.0008985
	Charge	45.4	37.6	16.99	0	0.09713	0.0407643
	Discharge	0	34.0	33.3	32.7	0.11070	0.0020389

Table S6. Parameters used in FEM simulations.

Name	Symbol	Value
Young Modulus of S	E_S	17.8 GPa
Young Modulus of LPSCl	E_{SE}	22 GPa
Poisson ratio of S	υ_S	0.32
Poisson ratio of LPSCl	υ_{SE}	0.3
Density of S	ρ_S	2 $g.cm^{-3}$
Density of LPSCl	ρ_{SE}	1.6 $g.cm^{-3}$
Hygroscopic coefficient of S_{micro}	β_H	$22.5.10^{-3}$ $m^3.kg^{-1}$
Hygroscopic coefficient of $S_{sub\text{-}micro}$	β_H	24.10^{-3} $m^3.kg^{-1}$
Molar mass of Li	M_m	7.10^{-3} $kg.mol^{-1}$

Table S7. Equations used in FEM simulations.

Name	Symbol
On external boundaries	$u = 0$
In the electrode	$\rho \frac{\partial^2 u}{\partial^2 t} = \nabla \mathbf{S} + F_v$<br>$S = C : \epsilon_{el}$<br>$C = C(E, v)$<br>$\epsilon_{el} = \epsilon - \epsilon_{inel}$<br>$\epsilon_{inel} = \epsilon_{HS}$<br>$\epsilon = \frac{1}{2}[(\nabla u)^T + \nabla u]$
Hygroscopic swelling in S particles	$\epsilon_{Hs} = \beta_H M_m C_{Li}$
Initial values	$u = 0$<br>$\frac{\partial u}{\partial t} = 0$

Table S8. Values used for energy density calculations. The SSE used is for the calculations is LPSCl.

Cell Parameters	Li || Sulfur	Li_2Si || Sulfur	100% Silicon || Li_2S	Li Metal || Li_2S	Anode-free || Li_2S
Nominal Voltage / V	1.7	1.7	1.4	1.7	1.7
Areal Capacity / mAh cm^{-2}	10	10	10	10	10
Positive Electrode / %	30	30	30	30	30
NP ratio	1.2	1.2	1.2	1.2	-
Negative Electrode Capacity / mAh g^{-1}	3500	860 (experimental)	3500	3500	-
Negative Electrode Density / g cm^{-3}	0.5	-	2.3	0.5	-
Positive Electrode Capacity / mAh g^{-1}	1600	1600	1000	1000	1000
Positive Electrode Density / g cm^{-3}	2	2	1.66	1.66	1.66
SSE Thickness / µm	30	30	30	30	30
SSE Density / g cm^{-3}	1.6	1.6	1.6	1.6	1.6
SSE Relative Density / %	85	85	85	85	85
Cu Foil Thickness / µm	10	10	10	10	10
Cu Foil Density / g cm^{-3}	8.9	8.9	8.9	8.9	8.9
Al Foil Thickness / µm	10	10	10	10	10
Al Foil Density / g cm^{-3}	2.7	2.7	2.7	2.7	2.7
Binder ratio / %	1	1	1	1	1
